# Green synthesis of Cu_2_S using garlic-derived sulfur: structural, morphological, optical, electrical, and photoresponse characterization with potential for thermoelectric applications

**DOI:** 10.1039/d4ra07771g

**Published:** 2025-02-03

**Authors:** M. M. Shahidi, M. Akbari, M. Mehrabani, A. Shams Khameneh, G. G. Welegergs, M. Aligholami, M. Maaza

**Affiliations:** a UNESCO-UNISA-ITL/NRF Africa Chair in Nanoscience and Nanotechnology, College of Graduate Studies, University of South Africa (UNISA) Muckleneuk Ridge, P.O. Box 392 Pretoria South Africa mahmoa@unisa.ac.za; b Department of Physics, Shahrood University of Technology Shahrood Iran

## Abstract

In this study, we present an environmentally friendly and sustainable green synthesis method for Cu_2_S thin films using sulfur sources derived from garlic (*Allium sativum* L.). This novel approach facilitates room-temperature sulfurization without the need for surfactants or artificial sulfur compounds, resulting in copper sulfide films with unique nanostructured morphologies. The synthesized Cu_2_S films were comprehensively characterized using FESEM, RBS, PIXE, Raman spectroscopy, Hall effect measurements, and DRS to evaluate their morphological, structural, optical, electrical, and photoresponse properties. FESEM analysis revealed well-defined nanostructures with a tubular sea coral-like morphology, while RBS and PIXE confirmed the successful formation and elemental composition of Cu_2_S with minimal impurities. Raman spectroscopy indicated the presence of both Cu_2_S and Cu_2_O phases, suggesting partial oxidation during synthesis. The optical properties assessed *via* DRS demonstrated a significant reduction in the band gap energy from 2.06 eV to 1.63 eV with increasing exposure time, highlighting the tunability of the material. Hall effect measurements confirmed p-type semiconductor behavior with enhanced electrical conductivity over time, and the photoresponse study showed a strong sensitivity to visible light, making the films suitable for optoelectronic applications. Compared to conventional methods, our green synthesis offers a low-cost, scalable, and environmentally friendly alternative, with potential applications in photovoltaics and thermoelectrics. This study highlights the potential of garlic-derived sulfur for sustainable nanomaterial synthesis and paves the way for its integration into energy conversion technologies.

## Abstract 2

This study presents a green synthesis of Cu_2_S thin films using garlic-derived sulfur at room temperature. The films exhibit unique properties, showing their potential for optoelectronic and thermoelectric applications.

## Graphical abstract

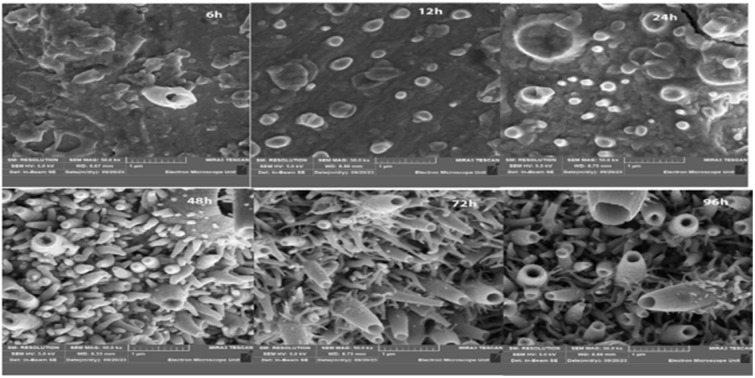

## 1. Introduction

Copper sulfides (Cu_*x*_S_*y*_) represent a diverse family of compounds, ranging from naturally occurring minerals to synthetic materials, with immense potential for various technological applications.^1–4^ This group includes prominent phases such as covellite (CuS)^5–7^ and chalcocite (Cu_2_S),^8,9^ along with a wide range of compositions, each exhibiting distinct crystallographic and electronic properties.^10–13^ Extensive research has been conducted on these materials, driven by their versatile applications in solid-state solar cells, electroconductive coatings, and energy storage devices.^3,14–19^ Our previous work^20^ introduced a pioneering green synthesis approach for these compounds, utilizing sulfur-rich organosulfur compounds naturally emitted from *Allium sativum* L. (garlic). This innovative method not only offers an environmentally friendly alternative but also enhances the physicochemical properties of the synthesized materials through the formation of unique nanostructures.

Conventional synthesis methods for Cu_*x*_S_*y*_ compounds often rely on surfactants,^21–23^ catalysts,^24^ or artificial sulfur sources,^25–28^ which can introduce contaminants and require significant energy input. In contrast, our novel approach leverages the naturally abundant sulfur compounds in garlic^20^ to achieve room-temperature sulfurization, resulting in a sustainable and eco-friendly process. The synthesized Cu_2_S films exhibit distinctive morphological and surface characteristics while maintaining favorable p-type semiconductor behavior, making them highly suitable for energy-related applications such as photovoltaics and thermoelectric devices.^17,18,20,29^

A variety of synthesis methods have been explored for Cu_2_S production, each resulting in distinct material properties. A comparative analysis of different synthesis approaches and their corresponding properties is presented in Table 1, highlighting the advantages of the green synthesis method employed in this study over conventional techniques.

**Table 1:** Comparison of synthesis methods and key properties of Cu_2_S thin films from different studies

Study	Synthesis method	Key properties	Band gap (eV)	Structural findings
Current study (garlic-derived sulfur method)	Green synthesis using garlic extract at room temperature	Unique nanostructure, p-type behavior, tunable bandgap	2.06–1.63	Cu_2_S phase with partial Cu_2_O oxidation
Singh *et al.* (2013)^30^	Chemical bath deposition (CBD) at 65 °C	Homogeneous, polycrystalline thin films with hexagonal structure	2.3	Spherical particles with spiky cone morphology
Usoh *et al.* (2014)^31^	Chemical bath deposition at room temperature	High absorbance, low transmittance	2.30–2.44	Amorphous structure with thickness-dependent properties
Dhanasekar *et al.* (2015)^32^	Chemical bath deposition	Optical band gap tunability with deposition time	2.8 (5 h), 2.7 (6 h)	Hexagonal structure with varying grain size
Ramírez-Esquivel *et al.* (2017)^33^	SILAR	Highly crystalline covellite CuS, good electrical properties	2.2–2.5	Polycrystalline structure without annealing
Shahzad *et al.* (2017)^34^	Vapor-phase sulfurization	2D β-Cu_2_S nanosheets with strong PL response	1.1	Thickness-controlled synthesis with phase-dependent properties
Savitskii *et al.* (2018)^35^	Exposure to gas-phase products of coal desulfurization	Large grain sizes, low resistivity, rough surface	1.91	Cubic structure with grain size 10–20 μm
Marimuthu *et al.* (2019)^36^	Hydrothermal synthesis	Flower and wall-like morphologies for different sulfur concentrations	2.02–2.24	Polycrystalline covellite phase
Hasan *et al.* (2019)^37^	Thermal evaporation with post-annealing	Optical constants vary with temperature	2.55–2.60	Amorphous structure at lower temperatures, crystallization with annealing
Aghad *et al.* (2024)^38^	Spin coating with annealing	CuS to Cu_2_S transition with annealing	1.63–1.87	Improved thermoelectric properties
Sabat *et al.* (2024)^39^	Chemical bath deposition with post-annealing	Enhanced crystallinity, tunable bandgap	1.66–2.81	Covellite to chalcocite phase transformation

Among the copper sulfide family, Cu_2_S stands out for its significant potential in thermoelectric applications. Recent research efforts have focused on enhancing the thermoelectric performance of Cu_2_S through various strategies, positioning it as a strong candidate for future energy conversion technologies. One promising strategy envolves compositional and structural modulation, where the introduction of second phases and Cu vacancies creates multiple lattice defects, such as nanoprecipitates and dislocations. These defects effectively suppress phonon transport, thereby reducing lattice thermal conductivity. With selenium (Se) doping, a high figure of merit (ZT) of approximately 1.6 at 873 K has been achieved, demonstrationg the material's enhanced performance.^40^ Another effective approach involves *in situ* phase separation, where natural minerals are introduced into Cu_1.8_S matrices, forming complex structures that further suppress phonon transmission. This method, combined with doping, results in a ZT value of 1.1 at 773 K, indicating improvements in both thermoelectric and mechanical properties.^41^ Doping approaches have also shown promise; for example, Mn-doped Cu_2−*x*_S films exhibit increased carrier concentration and mobility, enhancing their electrical properties. These films achieve a maximum power factor of 113.3 μW m^−1^ K^−2^, highlighting their potential for flexible thermoelectric generators.^42^ Additionally, a sulfur infusion process applied to 3D-printed Cu_2−*x*_S materials restores their thermoelectric properties, achieving a ZT of 1.0 at 780 K. This process not only optimizes the crystal structure but also provides a sustainable approach to thermoelectric material manufacturing.^43^ On a structural level, atomic and nanoscale order/disorder phenomena in copper-rich sulfides play a critical role in influencing the thermoelectric properties of Cu_2_S. These structural features impact the material's electronic and vibrational properties, which are essential for optimizing its thermoelectric performance.^44^ Despite Cu_2_S holds significant promise for thermoelectric applications, challenges remain in balancing its electrical and thermal properties to maximize efficiency. Further research into compositional adjustments, structural innovations, and advanced fabrication techniques is crucial to fully realize its potential.

Building on recent advancements, this study introduces a novel green synthesis methodology for Cu_2_S, utilizing sulfur sources derived from garlic. We provide on a detailed characterization of the synthesized Cu_2_S samples, exploring their structural, morphological, optical, electrical, and photoresponse properties to validate and further develop this environmentally friendly approach. The unique nanostructuration achieved through our synthesis method is hypothesized to influence phonon–electron interactions, potentially enhancing thermoelectric performance. Although direct thermoelectric measurements were not conducted, the observed electrical properties and Seebeck values indicate the material's potential suitability for thermoelectric applications. This study offers valuable insights into how these structural features may contribute to improved thermoelectric performance and highlights the importance of sustainable synthesis methods in advancing materials for future energy technologies.

## 2. Experimental and characterization methods

### 2.1 Synthesis and experimental methods

In continuation of our pursuit to validate and extend the findings of our prior work,^20^ the green synthesis of Cu_2_S thin films was conducted using sulfur sources derived from garlic (*Allium sativum* L.) at room temperature. High-purity copper (Cu, 99.99%) substrates with dimensions of 1.5 cm × 1.5 cm × 0.675 mm were used as the base material. The substrates were meticulously cleaned using an ultrasonic bath in sequential steps of isopropanol, acetone, and deionized water for 15 minutes each and subsequently dried with nitrogen gas to remove residual contaminants.

Fresh garlic cloves (50 g), sourced from local markets, were peeled and finely chopped using a standard electric blender to release bioactive volatile organosulfur compounds. The sulfurization process was performed in a closed glass chamber, where the Cu substrates were fixed to the container's cover using adhesive tape, maintaining a fixed distance of approximately 4 cm from the sulfur-emitting garlic sample. The substrates were exposed to the released sulfur vapors at ambient conditions for varying durations (6 h, 12 h, 24 h, 48 h, 72 h, and 96 h) to investigate the impact of exposure time on the structural and optical properties of the synthesized Cu_2_S films. Following the exposure period, the Cu substrates developed a visually distinct dark film layer, indicative of the formation of Cu_2_S. The samples were carefully removed and air-dried before further analysis.

This systematic approach enabled us to not only confirm the feasibility of achieving complete green sulfurization of copper surfaces but also to examine the consistency of the resulting films' properties over varying durations. Our experimental focus was on evaluating the repeatability of the synthesis process and conducting a comprehensive study of the structural, optical, and electrical properties of the films.

### 2.2 Characterization methods

To gain a comprehensively understanding of the morphological, structural, optical, electrical, and photoresponse characteristics of the synthesized copper sulfide films, we employed a range of advanced characterization techniques.

#### 2.2.1 Surface morphology and elemental analysis

The surface morphology of the samples was meticulously examined using field emission scanning electron microscopy (FESEM). High-resolution images obtained through this technique provided a detailed assessment of the nanostructures and surface features formed *via* the green sulfurization process. Prior to imaging, the samples were carbon-coated using an evaporation coater (MED 010, Balzers Union, Liechtenstein) to enhance conductivity. Morphological analysis was performed with a Nova NanoSEM (Oxford Instruments, Oxfordshire, United Kingdom), equipped with an Oxford X-Max detector, operating at 20 kV, with a 20 mm^2^ detector area and a 6 mm working distance.

For elemental composition and spatial distribution analysis, two complementary techniques were employed.

#### 2.2.2 Rutherford backscattering spectrometry (RBS)

RBS was conducted using ^4^He ions at an incident energy of 3.077 MeV, with a scattering angle of 150° and a detector resolution (FWHM) of 23 keV. The data were analyzed with SIMNRA software, which confirmed the successful formation of Cu_2_S nanostructures and providing insights into layer composition and thickness.

#### 2.2.3 Proton-induced X-ray emission (PIXE)

PIXE analysis was performed using a 3.0 MeV proton beam with a current ranging from 50 to 100 pA. A PGT Si(Li) detector with an energy resolution of approximately 160 eV was used to capture the data, which was processed and analyzed using GEOPIXE software. This technique allowed precise quantification and mapping of the elemental distribution within the samples.

#### 2.2.4 Optical properties

The optical properties of the copper sulfide films were evaluated using diffuse reflectance spectroscopy (DRS) across the wavelength range of 200–2500 nm. An Ocean Optics Maya 2000 Pro spectrometer, coupled with a DH-2000-BAL UV-vis-NIR light source, was employed for these measurements. DRS analysis facilitated the determination of optical absorption characteristics and estimation of bandgaps, which are crucial for understanding the materials' photovoltaic and photoresponsive properties.

#### 2.2.5 Structural properties

Raman spectroscopy was utilized to investigate the structural properties of the synthesized films, providing valuable insights into their crystallographic structures and phase compositions. These analyses helped establish correlations between the synthesis conditions and the resulting material phases. Measurements were carried out using a Witec Confocal Raman Microscope (alpha300) under 532 nm laser excitation, enabling precise characterization of the material's structural integrity.

#### 2.2.6 Electrical properties

The electrical characteristics of the copper sulfide films were examined through Hall effect measurements, focusing on the temperature-dependent variations. This analysis was essential for understanding the semiconductor behavior of the films, which is critical for their potential application in electronic devices. Measurements were conducted using an Ecopia (HMS 7000) system within a temperature range of 300 to 420 K.

#### 2.2.7 Photoresponse properties

The photoresponse properties of the films were assessed under various colored light sources to evaluate their suitability for photodetector applications. This analysis measured the sensitivity and response times of the films across different wavelengths, providing insights into their potential utility in light detection and conversion into electrical signals using the Autolab Potentiostat (CH Instrument, USA) system.

Collectively, these sophisticated characterization techniques form the backbone of our investigation, providing a holistic understanding of the copper sulfide films synthesized *via* green sulfurization. This comprehensive approach ensures a thorough analysis of the material's properties, paving the way for future electronics, photovoltaics, and photodetection applications.

## 3. Results and discussion

### 3.1 FESEM

**Fig. 1:**
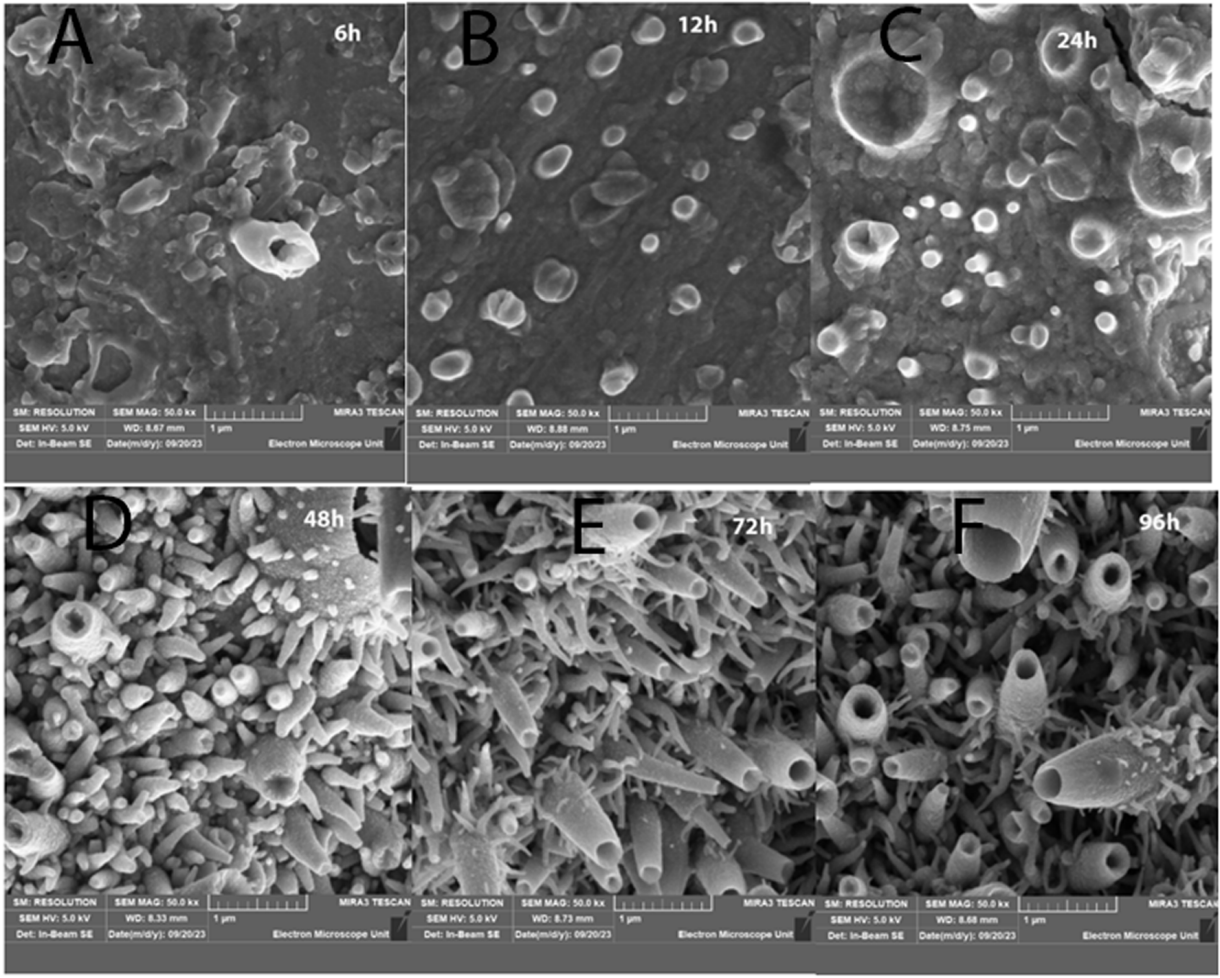
Surface morphology of Cu substrate exposed to garlic-derived organosulfur compounds for varying durations observed *via* FESEM at 1 μm resolution. Images depict samples after (a) 6 h, (b) 12 h, (c) 24 h, (d) 48 h, (e) 72 h, and (f) 96 h exposure times. Prolonged exposure (48 h, 72 h, and 96 h) leads to pronounced nanostructure formation, resembling tubular sea coral reefs, indicating the progressive impact of time-dependent sulfurization on nanostructure development.

Fig. 1 presents the surface morphology and nanoparticle growth observed through FESEM images at a resolution of 1 μm, following the methodology established in previous studies. The images capture the surface of the Cu substrate after exposure to organosulfur emissions from *Allium sativum* (garlic) at room temperature for various durations: 6 h, 12 h, 24 h, 48 h, 72 h, and 96 h. Notably, after 48 h, 72 h, and 96 h, the surface exhibits a pronounced and extensive nanostructure formation. These samples display a morphology reminiscent of a tubular sea coral reef, with intricate details becoming more apparent with longer exposure times. This unique structural growth mirrors observations from previous studies, reinforcing the impact of time-dependent exposure on nanostructure development.^20,45,46^

### 3.2 RBS

**Fig. 2:**
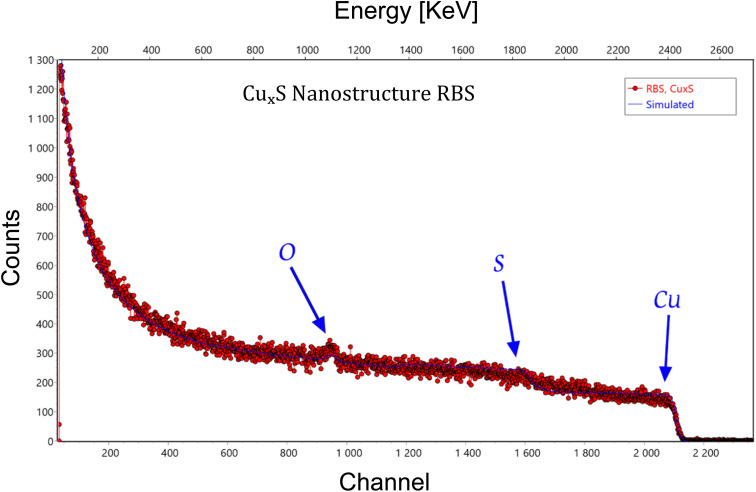
This RBS spectrum illustrates the formation of Cu_2_S nanostructures, using ^4^He ions at 3.077 MeV. The experimental data (solid line) and the simulated fit (dashed line) show clear peaks for copper (Cu) and sulfur (S), with oxygen (O) likely from surface oxidation. The high-energy edge corresponds to the Cu surface layer, followed by the sulfur peak. This analysis confirms the layer composition and thickness, validating the successful synthesis of Cu_*x*_S nanostructures.

The RBS spectrum of Cu_2_S nanostructures was obtained using ^4^He ions at an incident energy of 3.077 MeV. The experimental data (solid line) were analyzed using SIMNRA software, with the simulated fit (dashed line) shown for comparison. Distinct peaks corresponding to copper (Cu) and sulfur (S) are clearly visible, with the high-energy edge indicating the Cu surface layer, followed by the S peak at lower energies due to its lighter atomic mass. Oxygen (O) was also detected, likely originating from surface contamination or oxidation. The experimental conditions included a scattering angle of 150°, and a detector resolution (FWHM) of 23 keV. The spectrum confirms the successful formation of Cu_2_S nanostructures, with the simulation data closely matching the experimental data, validating the layer thickness and composition, as shown in Fig. 2. The first layer, with a thickness of 10.21 × 10^15^ at. per cm^2^, consists of Cu, S, and O, while the second, thicker layer at 5235.73 × 10^15^ at. per cm^2^, primarily contains Cu and S. Beneath these layers, the bulk copper substrate (layer 3) extends to 1.178 × 10^6^ × 10^15^ at. per cm^2^.

### 3.3 PIXE

**Fig. 3:**
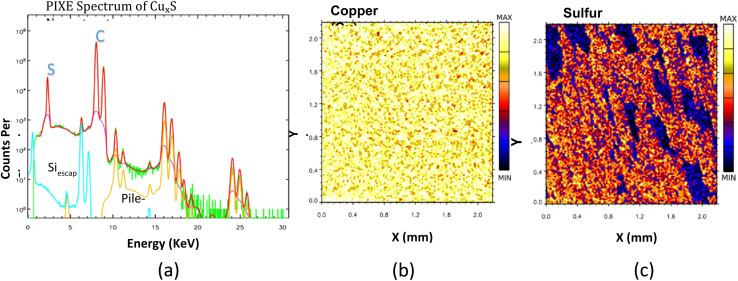
PIXE analysis of Cu_2_S nanostructures. (a) PIXE spectrum showing characteristic X-ray emission peaks for copper (Cu Kα) and sulfur (S Kα). (b) Elemental mapping of copper (Cu), illustrating uniform spatial distribution across the nanostructure surface. (c) Elemental mapping of sulfur (S), confirming homogeneous distribution consistent with the copper distribution.

Proton-induced X-ray emission (PIXE) analysis was performed to determine the elemental composition and spatial distribution of copper and sulfur within the synthesized Cu_2_S nanostructures. The PIXE spectrum (Fig. 3a) displays distinct peaks corresponding to the copper (Cu Kα) and sulfur (S Kα) X-ray emission lines, confirming the successful presence of these elements in the sample. The analysis was conducted using a 3.0 MeV proton beam with a current of 50–100 pA and a PGT Si(Li) detector with an energy resolution of approximately 160 eV. To minimize artifacts, such as pile-up effects, the measurement conditions were carefully optimized, ensuring accurate quantification of the elemental composition.

Elemental mapping was utilized to visualize the distribution of copper and sulfur within the nanostructures. The copper elemental map (Fig. 3b) demonstrates a uniform spatial distribution, with brighter regions indicating areas of higher copper concentration. Similarly, the sulfur elemental map (Fig. 3c) reveals a consistent distribution of sulfur across the sample surface. The even distribution of both elements confirms the homogeneity of the synthesized Cu_2_S nanostructures, verifying the effectiveness of the green synthesis process.

### 3.4 Optical properties

**Fig. 4:**
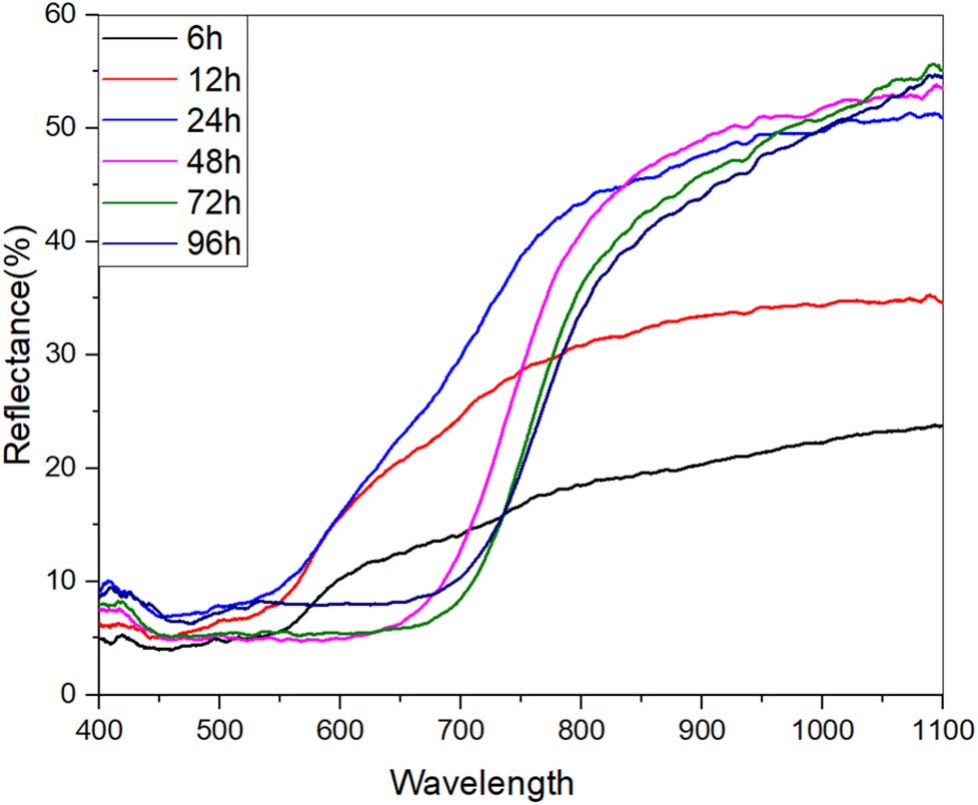
Diffuse reflectance spectroscopy (DRS) spectra of Cu_2_S samples synthesized at different exposure times (6 h, 12 h, 24 h, 48 h, 72 h, 96 h) over 400–1100 nm. Low reflectance within the visible range (400–600 nm) indicates effective light absorption, while a sharp increase beyond 700 nm suggests a shift towards the near-infrared region. The rise between 600 and 700 nm marks the absorption threshold, reflecting the time-dependent evolution of optical properties.

The optical properties of the materials were evaluated using diffuse reflectance spectroscopy (DRS) in the UV-visible range. Fig. 4 shows the reflection spectra of the prepared samples over the wavelength range of 400–1100 nm. The data reveal that the reflectance for the 24 h, 48 h, 72 h, and 96 h samples increases significantly beyond 700 nm, indicating a shift in optical response as the wavelength approaches the near-infrared region.

In the visible region (400–600 nm), all samples exhibit relatively low reflectance, with maximum values around 15%, suggesting efficient light absorption in this region-an essential characteristic for potential applications in photovoltaic and optoelectronic devices. Additionally, a gradual increase in reflectance is consistently observed between 600 and 700 nm, which likely corresponds to the material's absorption threshold. This feature indicates the transition from strong absorption in the visible range to increased reflectivity at longer wavelengths, further confirming the time-dependent evolution of the material's optical properties as a function of the synthesis duration.

**Fig. 5:**
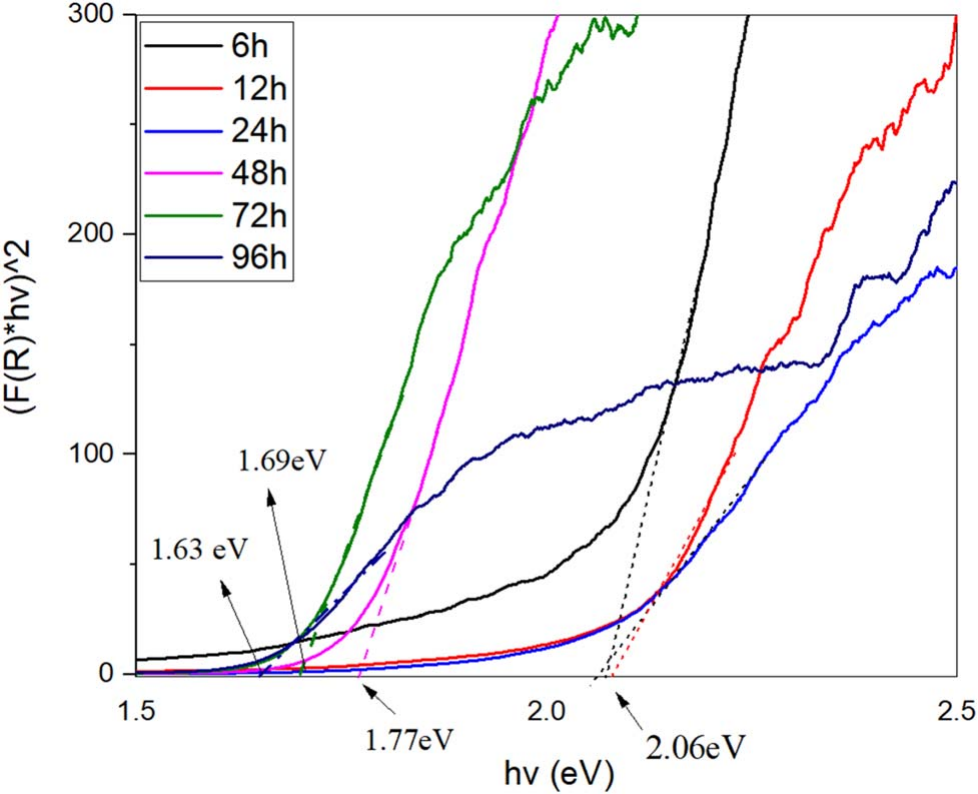
Band gap energy estimation of Cu_2_S samples synthesized at different exposure times (6 h, 12 h, 24 h, 48 h, 72 h, 96 h) using the Kubelka–Munk function. The plots of (*F*(*R*) × *hν*)^2^*vs.* photon energy (*hν*) show a decrease in band gap from 2.06 eV (6 h) to 1.63 eV (96 h), indicating changes in the material's electronic structure with extended synthesis.

In Fig. 5, the band gap energy of the samples was evaluated using the Kubelka–Munk equation by plotting (*F*(*R*) × *hν*)^2^ against *hν* and fitting the linear portion of the curves. The Kubelka–Munk function is expressed as:1
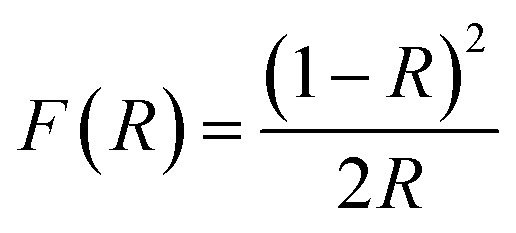
where *R* represents the reflectance of the surface of the thin film. This approach allows for the estimation of the optical band gap energy by analyzing the intersection of the linear fit with the energy axis.^20,47,48^

As the exposure time increased, a significant reduction in the band gap energy of the samples was observed. The 6 h sample exhibited a band gap of approximately 2.06 eV, whereas the 96 h sample showed a reduced band gap of around 1.63 eV. This time-dependent decrease in the band gap suggests that prolonged exposure to the sulfur source promotes changes in the electronic structure of Cu_2_S, possibly due to increased nanostructuration or the formation of more complex bonding environments within the material.

The reduction in band gap with increased exposure time indicates enhanced electronic interactions or changes in crystallinity, which could influence the optical and electrical properties of the material. This trend is particularly relevant for applications in optoelectronics and thermoelectrics, where precise band gap tuning is crucial for optimizing performance. A summary of the relationship between synthesis duration and band gap energy is provided in Table 2.

**Table 2:** The parameters extracted from optical properties

Sample (hours)	6 h	12 h	24 h	48 h	72 h	96 h
Band gap (eV)	2.06	2.06	2.03	1.77	1.69	1.63

### 3.5 Raman study

**Fig. 6:**
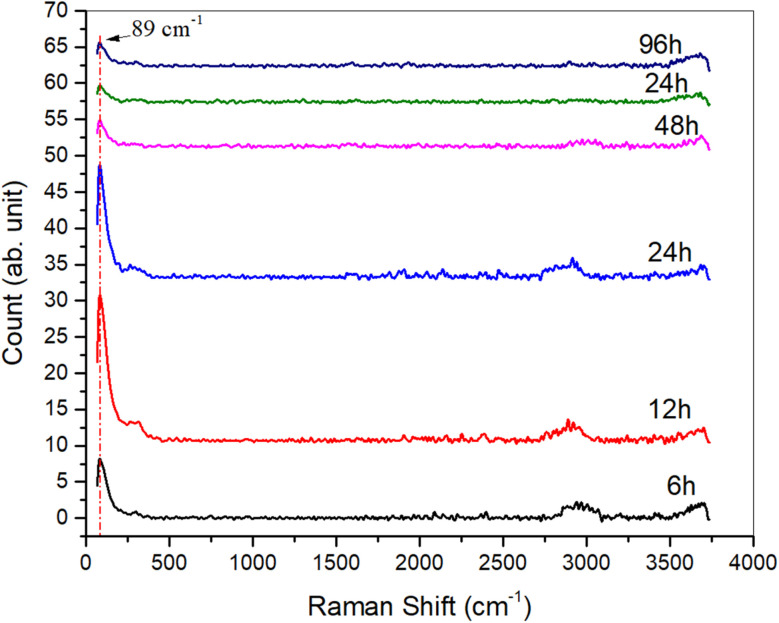
Raman spectra of Cu_2_S samples synthesized at varying exposure times (6 h, 12 h, 24 h, 48 h, 72 h, 96 h). The absence of characteristic Cu_2_S peaks indicates an amorphous phase, while observed Cu_2_O peaks suggest surface oxidation, likely affecting the material's optical and electrical properties.

To further investigate the structural properties of the samples, Raman spectra were obtained, as shown in Fig. 6. The absence of the characteristic Raman peaks typically associated with crystalline copper sulfide (Cu_2_S) suggests that the Cu_2_S phase in these samples is predominantly amorphous. This amorphous nature is likely due to the specific synthesis conditions, which hinder the formation of well-defined crystalline structures.

Interestingly, the observed peaks in the Raman spectra are attributed to the presence of a Cu_2_O (cuprous oxide) thin layer, which likely formed during the synthesis process. The Cu_2_O peaks indicate a degree of surface oxidation of the Cu_2_S, possibly occurring during exposure to ambient conditions or as part of the chemical interaction with the sulfur source. This structural composition, combining amorphous Cu_2_S with a Cu_2_O surface layer, could influence both the optical and electrical properties of the material, and may play a role in its thermoelectric behavior.^49^ These findings highlight the importance of further investigating the interplay between synthesis conditions and structural formation to better understand their impact on the overall properties of the material.

### 3.6 Photo response study

**Fig. 7:**
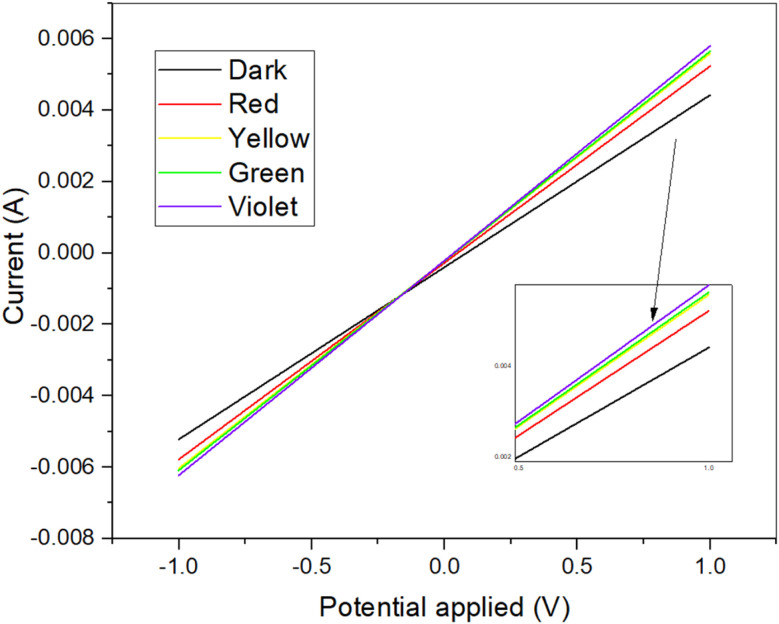
Current–voltage (*I*–*V*) characteristics of the 96 h Cu_2_S sample measured in the dark and under illumination with different wavelengths (red, yellow, green, violet). The linear curves indicate consistent ohmic behavior, with slight variations in resistance under light exposure. The increased current response with shorter wavelengths demonstrates the material's sensitivity to higher-energy light, suggesting potential for optoelectronic applications.

Fig. 7 presents the current–voltage (*I*–*V*) characteristics of the 96 h sample, measured both in the dark and under illumination at various light wavelengths. The *I*–*V* measurements were performed on the thin layers of Cu_2_S between four contacts, within a voltage range of −1 V to 1 V. The results demonstrate a clear ohmic behavior across all conditions, with only slight variations in resistance observed under different light spectra, including black, red, yellow, green, and violet. As expected, the optical response increases with decreasing wavelength (*i.e.*, increasing photon energy), highlighting the material's sensitivity to higher-energy light.

This consistent ohmic behavior across all measurements confirms the reliability of the Hall effect tests, further validating the electrical properties of the sample. The response to varying light wavelengths suggests that Cu_2_S exhibits potential for applications in optoelectronic devices, as its electrical characteristics are influenced by light exposure, particularly in the visible range.^50^

### 3.7 Electrical properties

**Fig. 8:**
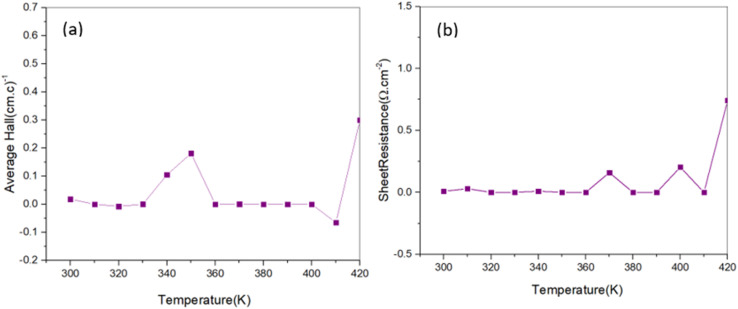
Temperature-dependent electrical properties of the 96 h Cu_2_S sample. (a) Hall coefficient measurements confirm p-type conductivity, indicating holes as the dominant charge carriers. (b) Sheet resistance increases slightly at 420 K, reflecting typical semiconductor behavior where higher temperatures lead to increased carrier scattering and reduced mobility. These insights are crucial for assessing the material's thermoelectric potential.

The electrical properties of the 96 h sample were evaluated at different temperatures using the Hall effect apparatus. The obtained parameters are crucial for further exploration of its thermoelectric potential. Fig. 8a illustrates the majority carrier concentration, where the positive Hall coefficient confirms that the Cu_2_S thin films exhibit p-type semiconductors behavior, indicating that holes are the dominant charge carriers in the material.

Fig. 8b presents the sheet resistance of the 96 h sample measured across various temperatures. The data reveal a slight increase in sheet resistance at 420 °C, suggesting that the electrical resistivity of the material rises with temperature. This behavior is characteristic of semiconductors, where increased thermal energy leads to increased scattering of charge carriers, thereby reducing their mobility. These findings provide valuable insights into the temperature-dependent electrical properties of Cu_2_S, which are essential for evaluating its potential in thermoelectric applications.^51^

### 3.8 Seebeck coefficient

**Fig. 9:**
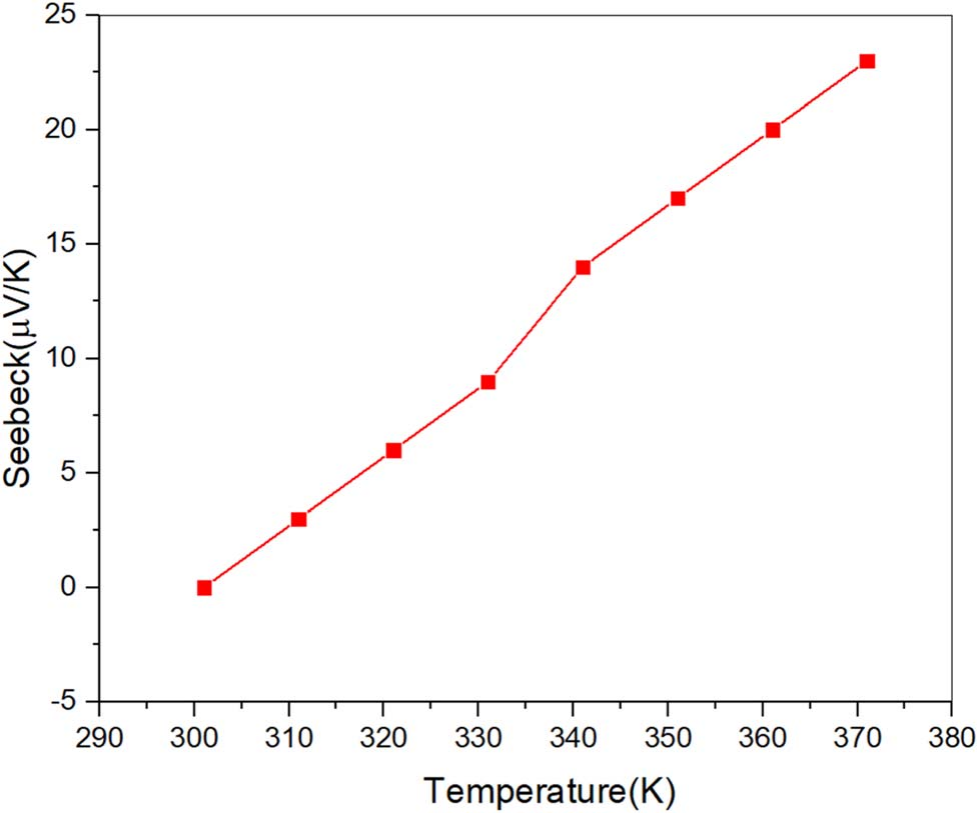
Temperature-dependent Seebeck coefficient of the 96 h Cu_2_S sample. The positive Seebeck values across all temperatures confirm p-type behavior, with a peak value of approximately 20 μV K^−1^ at higher temperatures. Variations in the coefficient suggest the influence of polymorphic transitions, indicating the material's potential for thermoelectric applications.

Fig. 9 presents the Seebeck coefficient measurements for the examined samples, showing similar average values consistent with those measured at room temperature. Notably, the 96 h sample exhibits positive Seebeck coefficient values across the entire temperature range, confirming its p-type semiconductor behavior. The positive Seebeck coefficient indicates that holes are the dominant charge carriers in the material.

The 96 h thin film shows a higher Seebeck coefficient of approximately 20 μV K^−1^, which is significant for thermoelectric applications. Furthermore, distinct boundaries and variations in the Seebeck coefficient are observed at specific temperature ranges, corresponding to different polymorphic forms of Cu_2_S. These transitions in polymorphic structure likely influence the material's thermoelectric properties, resulting in changes in the Seebeck coefficient with temperature. This correlation between structural phase transitions and thermoelectric performance highlights the material's potential for optimized energy conversion at varying temperatures.^52,53^

## 4. Conclusion

In this study, we successfully demonstrated a sustainable, green synthesis method for Cu_2_S thin films using garlic-derived sulfur. This environmentally friendly approach eliminates the need for surfactants or artificial sulfur sources, offering a low-cost and scalable alternative to conventional methods. Comprehensive characterization revealed that the synthesized films exhibit unique nanostructured morphologies, tunable optical properties with a band gap reduction from 2.06 eV to 1.63 eV, p-type semiconductor behavior, and enhanced electrical conductivity with prolonged exposure to sulfur vapors. The observed positive Seebeck coefficient confirms the potential thermoelectric applicability of the films.

A key advancement of this work lies in the demonstration of a completely green sulfurization process at room temperature, which contrasts with traditional synthesis methods that often require high temperatures, surfactants, or hazardous chemicals. Compared to previously reported methods, our approach enhances material uniformity and phase purity, while significantly reducing environmental impact and synthesis complexity.

Our findings highlight that the unique nanostructures formed through this green synthesis method influence phonon–electron interactions, potentially enhancing the material's thermoelectric performance. This study not only contributes to the growing field of sustainable nanomaterial synthesis but also provides valuable insights into the structure–property relationships of Cu_2_S thin films for applications in photovoltaics, thermoelectrics, and optoelectronic devices.

Future work will focus on further optimizing synthesis parameters, including exposure duration and environmental conditions such as vacuum or inert atmospheres, to achieve improved material properties and stability. Additionally, advanced characterization techniques, such as grazing incidence X-ray diffraction (GIXRD), will be employed to gain deeper insights into the structural phases. Moreover, direct thermoelectric performance evaluations will be conducted to establish the practical viability of these green-synthesized Cu_2_S thin films in real-world energy applications.

## Data availability

The original data of the study are included in the article. Further inquiries can be directed to the corresponding author.

## Author contributions

M. M. Shahidi: investigation, validation, methodology, writing – original draft. M. Akbari: investigation, validation, methodology, writing – review & editing, supervision, conceptualization. M. Mehrabani: investigation, validation, methodology. A. Shams Khameneh: investigation, validation, methodology. G. G. Welegergs: conceptualization, investigation, validation. M. Aligholami: methodology, validation. M. Maaza: supervision, conceptualization, investigation, validation.

## Conflicts of interest

There are no conflicts to declare.
