# Correction: Green synthesis of Cu_2_S using garlic-derived sulfur: structural, morphological, optical, electrical, and photoresponse characterization with potential for thermoelectric applications

**DOI:** 10.1039/d5ra90031j

**Published:** 2025-03-31

**Authors:** M. M. Shahidi, M. Akbari, M. Mehrabani, A. Shams Khameneh, G. G. Welegergs, M. Aligholami, M. Maaza

**Affiliations:** a UNESCO-UNISA-ITL/NRF Africa Chair in Nanoscience and Nanotechnology, College of Graduate Studies, University of South Africa (UNISA) Muckleneuk Ridge, P. O. Box 392 Pretoria South Africa mahmoa@unisa.ac.za; b Department of Physics, Shahrood University of Technology Shahrood Iran

## Abstract

Correction for ‘Green synthesis of Cu_2_S using garlic-derived sulfur: structural, morphological, optical, electrical, and photoresponse characterization with potential for thermoelectric applications’ by M. M. Shahidi *et al.*, *RSC Adv.*, 2025, **15**, 3406–3415, https://doi.org/10.1039/D4RA07771G.

The authors regret the omission of an acknowledgement in the original article. This acknowledgement is given below.

We appreciate iThemba LABS/NRF for providing the facilities to conduct the experiments, and we extend our special thanks to Dr Christopher Mtshali for his valuable collaboration in RBS and PIXE characterization.

The Royal Society of Chemistry apologises for these errors and any consequent inconvenience to authors and readers.

